# The Dose–Response Relationship Between Tobacco Education Advertising and Calls to Quitlines in the United States, March–June, 2012

**DOI:** 10.5888/pcd12.150157

**Published:** 2015-11-05

**Authors:** Kevin C. Davis, Robert L. Alexander, Paul Shafer, Nathan Mann, Ann Malarcher, Lei Zhang

**Affiliations:** Author Affiliations: Robert L. Alexander, Jr, Ann Malarcher, Lei Zhang, Office on Smoking and Health, Centers for Disease Control and Prevention, Atlanta, Georgia; Paul Shafer, Nathan Mann, RTI International, Research Triangle Park, North Carolina.

## Abstract

**Introduction:**

We estimated changes in call volume in the United States in response to increases in advertising doses of the *Tips From Former Smokers* (*Tips*) campaign, the first federal national tobacco education campaign, which aired for 12 weeks from March 19 to June 10, 2012. We also measured the effectiveness of ad taglines that promoted calls directly with a quitline number (1-800-QUIT-NOW) and indirectly with a cessation help website (Smokefree.gov).

**Methods:**

Multivariate regressions estimated the weekly number of calls to 1–800-QUIT-NOW by area code as a function of weekly market-level gross rating points (GRPs) from CDC’s *Tips* campaign in 2012. The number of quitline calls attributable solely to *Tips* was predicted.

**Results:**

For quitline-tagged ads, an additional 100 television GRPs per week was associated with an increase of 89 calls per week in a typical area code in the United States (*P* < .001). The same unit increase in advertising GRPs for ads tagged with Smokefree.gov was associated with an increase of 29 calls per week in any given area code (*P* < .001). We estimated that the *Tips* campaign was responsible for more than 170,000 additional calls to 1–800-QUIT-NOW during the campaign and that it would have generated approximately 140,000 additional calls if all ads were tagged with 1–800-QUIT-NOW.

**Conclusion:**

For campaign planners, these results make it possible to estimate 1) the likely impact of tobacco prevention media buys and 2) the additional quitline capacity needed at the national level should future campaigns of similar scale use 1–800-QUIT-NOW taglines exclusively.

## Introduction

Telephone quitlines are a core element of comprehensive state tobacco control programs, and their promotion has been shown to encourage quit attempts and improve smoking cessation outcomes (1,2). Quitlines provide services to smokers, including counseling, free or reduced-price nicotine replacement therapy, and referrals to other cessation resources (3,4). All 50 US states and the District of Columbia operate quitlines. All state quitlines can be reached by calling 1–800-QUIT-NOW, which transfers callers to their state quitline. The Centers for Disease Control and Prevention (CDC) provides supplemental funding for state quitlines, and the National Cancer Institute (NCI) manages the portal function. Fifteen states (including large states such as California, New York, and Florida) use and promote alternate quitline numbers that connect callers directly to their state quitlines.

Although quitlines have been shown to increase smokers’ success in quitting (1,2), nationally only 1% to 2% of smokers use a state quitline in any given year, with state rates ranging from 0.1% to 6% (5). Quitlines’ reach in assisting smokers with cessation can be enhanced by interventions to increase their use. Media campaigns to prevent tobacco use are used by state tobacco control programs to promote quitline use, and multiple studies have demonstrated their effects on increased calls to quitlines in the United States (6–8) and in other countries (9–13). Although most of this research focuses on the effects of increased dosing of television ads at the state level, at least one study showed that television and radio ads increased quitline call volume, with television advertising having the largest effect (14,15).

In 2012, CDC launched the first federally funded national tobacco education campaign in the United States: *Tips From Former Smokers* (*Tips*). This campaign ran for 12 weeks from March 19 to June 10, 2012, and consisted of television ads on national cable networks and selected local networks. Although television was the primary media channel, radio ads (limited to 18 local markets), video ads on websites such as YouTube, online banners and displays, print ads, movie theater ads, and out-of-home ads (ie, billboards, bus shelters, and outdoor venues) were also used. *Tips* campaign ads featured graphic and emotional true stories about the severe health consequences of smoking told by former smokers to encourage current smokers to quit (16). These strategies were based on research showing that smokers are more likely to remember graphic or emotional antismoking messages than any other type of message (17,18). Such messages are also more effective at promoting cessation than other types of ads (19,20). All *Tips* television ads and other campaign materials can be found at the Tips campaign website, www.cdc.gov/Tips.

Approximately one-third of *Tips* television ads and all radio ads were tagged with 1–800-QUIT-NOW, which was read aloud in both types of ads. The remaining two-thirds of television ads were tagged with the Smokefree.gov website address. Ads tagged with 1–800-QUIT-NOW aired primarily during weekdays before 8 pm to correspond with the operating hours of some state quitlines. This pattern in tagging was adopted to ensure that state quitlines were not overwhelmed with a greater call volume than could be reasonably processed. The *Tips* campaign also included Spanish and English versions of one ad that was placed on Hispanic television and tagged with 1–800-QUIT-NOW.

A study in 2013 showed that the *Tips* campaign was successful in generating an increase in the prevalence of quit attempts by US smokers (21). In addition, initial trend data on 1–800-QUIT-NOW call volume in 2012 showed that the *Tips* campaign increased the number of calls to 1–800-QUIT-NOW (22). However, this initial research was primarily aimed at assessing pre–post changes in quitline call volume, because measures of the distribution of *Tips* campaign advertising were not available at the time of the analysis.

Although the evidence on the effectiveness of media in promoting calls to quitlines is well-established, several gaps in this research remain. The evidence is predominantly based on state campaigns. To our knowledge, no study has examined the effect of mass media campaigns to prevent tobacco use on nationwide quitline calls in the United States. In addition, research has not assessed the effects of ad taglines for promoting calls to quitlines. The objective of this study was to evaluate the impact of the *Tips* campaign on nationwide quitline use by quantifying the relationship between the volume of calls to quitlines from each telephone area code and the level of *Tips* campaign advertising in each television and radio market area. As such, we extended the state-focused evidence base by being the first study to evaluate the impact of increased dosing of a national tobacco prevention campaign on nationwide quitline calls in the United States. These relationships were examined separately for ads tagged with 1–800-QUIT-NOW and those tagged with Smokefree.gov. In addition, we expanded on the limited evidence of effectiveness by media channel by quantifying the associations between measures of television advertising and measures of radio advertising for the *Tips* campaign.

## Methods

### Data

The outcome variable was the number of calls per week by area code to 1–800-QUIT-NOW. Call volume data for 1–800-QUIT-NOW were obtained in collaboration with NCI and included weekly call totals during the 4 weeks before the *Tips* campaign through 4 weeks after the campaign (20 weeks in total). The data included total call volume, not unique callers, including 4,780 observations for aggregated weekly calls from 239 unique area codes. The *Tips* campaign exposure variables were weekly media market-level *Tips* gross rating points (GRPs) for television and radio advertising. GRPs measure the relative “dose” of advertising delivered to a given audience (eg, general adults, Hispanic adults) in a given media market and time period. GRPs are defined as the product of the percentage of the audience that is exposed (ie, audience reach) and the frequency with which that exposure occurs (ie, the number of times ads are aired). For example, if 75% of a media market’s television audience is exposed to *Tips* ads twice in a week, the television GRP for that market in that week would equal 150 (75 × 2) (15). GRP data are based on Nielsen television and radio ratings data for programs during which *Tips* ads aired.

The analysis included separate variables for television GRPs dedicated to ads tagged with 1–800-QUIT-NOW and ads tagged with Smokefree.gov. Although we anticipated that ads tagged with the quitline number would increase calls the most, smokers could also see the 1–800-QUIT-NOW number on Smokefree.gov. To account for this potential indirect influence on call volume, we included a separate variable for GRPs for ads tagged with the Smokefree.gov address. We also included a separate variable for GRPs for *Tips* ads aired on Hispanic television. Because Hispanic and general audience GRPs are calculated from different audience bases, they were measured separately and could not be summed.

Weekly media market GRPs were merged to the call volume data by linking the area code for each call to a designated market area (DMA) that geographically defines each television and radio market. When the area code overlapped multiple DMAs, we assigned the area code to the DMA that had the largest proportion of the area code’s adult population. Three area codes did not have a matching DMA in the United States. These area codes, from which 3,257 calls were made (<1% of total 481,856 calls), were excluded from analysis.

### Analysis

We first plotted total calls by total population-weighted weekly television and radio GRPs to provide a basic description of the relationship between advertising GRPs and calls. Next, we estimated multivariate linear regressions of weekly area code–level calls as a function of DMA-level weekly GRPs for television ads tagged with 1–800-QUIT-NOW, GRPs for television ads tagged with Smokefree.gov, GRPs for radio ads, GRPs for Hispanic television ads tagged with 1–800-QUIT-NOW, and numerous control variables. Each GRP variable was scaled to provide coefficients that yielded the increase in weekly area code calls given an increase of 100 weekly GRPs in a given market. Although coefficients on each of the main television-specific GRP variables (1-800-QUIT-NOW and Smokefree.gov) are comparable with each other, they are not comparable with the coefficient for radio GRPs or the coefficient for Hispanic television GRPs, because the audience bases for radio GRPs and Hispanic television GRPs are different from those for general audience television GRPs. To facilitate comparability between the effects of each type of advertising, we computed an elasticity value for each GRP variable. Elasticity is a measure of the percentage change in calls for a given percentage change in GRPs. For example, if the GRP elasticity is 0.2, a 10% increase in advertising GRPs would yield a 2% increase in weekly calls.

Our model controlled for area code population (in 100s of thousands), the percentage of the DMA population that was African American or Hispanic, the percentage of the DMA population that had a bachelor’s degree or higher, and the median household income in the DMA. We also controlled for DMA-level adult smoking prevalence in 2012, derived by aggregating recently published (23) county-level estimates of smoking prevalence for the DMA, weighted by county population. To account for fixed state-level differences in tobacco control policies and taxes, we included separate variables for each state. We also included a linear time trend to account for changes in call volume over time that may have been independent of the *Tips* campaign. Finally, we controlled for additional media buys for *Tips* television ads that were purchased separately by states with their own funds. GRPs for these supplemental ad buys were included as a separate control variable.

We also considered alternative model specifications that included quadratic terms to account for a possible nonlinear relationship with GRPs and state-specific tobacco policy variables (instead of state fixed effects) consisting of state cigarette taxes and state tobacco control program funding. However, comparisons of model fit statistics and estimated variance inflation factors, the Akaike information criterion, and the Bayesian information criterion (24) indicated that the simple linear model with state fixed effects fit the data best.

Using our regression model results, we then performed post-estimation predictions under a “no campaign” assumption to estimate the total additional calls to 1–800-QUIT-NOW that were attributable to the *Tips* campaign. The “no campaign” scenario assumed that all CDC- and state-funded *Tips* campaign GRPs were zero, because *Tips* ads would not have been available to states if not for the existence of the national campaign. We also used this procedure to predict the number of campaign-attributable calls that would have been made had all television ads been tagged with 1–800-QUIT-NOW.

## Results

During the *Tips* campaign, 363,656 calls were placed to 1–800-QUIT-NOW, an average of 30,304 calls per week; an average of 14,775 per week was made during the 4 weeks before and after the campaign (Figure). Variation in weekly call volume corresponded to patterns in weekly television GRPs for ads tagged with 1–800-QUIT-NOW and Smokefree.gov. Because radio ads aired only in 18 markets, overall radio exposure was relatively low.

**Figure:**
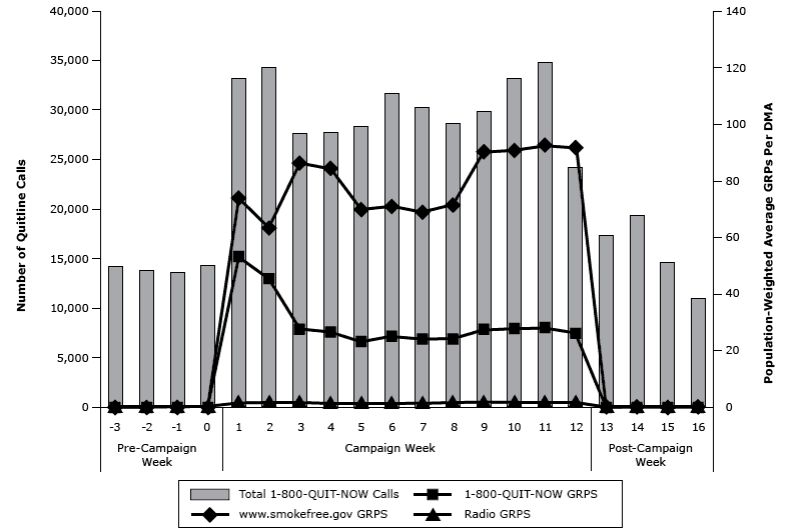
Weekly total calls to 1–800-QUIT-NOW and total population-weighted *Tips* campaign television and radio GRPs, February 4–July 8, 2012. Abbreviations: DMA, designated market area; GRP, gross rating point. **Table:** 

Week	Total 1-800-QUIT-NOW Calls	Weighted Total Gross Rating Points	1-800-QUIT-NOW Gross Rating Points	www.smokefree.gov Gross Rating Points	Weighted Radio Gross Rating Points
**Pre-campaign week**					
−3	14,230	0.0	0.0	0.0	0.0
−2	13,747	0.0	0.0	0.0	0.0
−1	13,617	0.0	0.0	0.0	0.0
0	14,338	0.0	0.0	0.0	0.0
**Campaign week**					
1	33,141	127.3	53.2	74.1	1.9
2	34,279	108.4	45.3	63.2	1.9
3	27,639	113.7	27.5	86.2	1.9
4	27,752	111.1	26.6	84.5	1.6
5	28,345	92.9	23.1	69.8	1.6
6	31,698	96.0	25.1	70.9	1.6
7	30,237	92.9	24.0	68.9	1.6
8	28,606	95.5	24.1	71.4	1.8
9	29,882	117.8	27.5	90.3	1.9
10	33,136	118.3	27.7	90.6	1.9
11	34,763	120.6	28.1	92.5	2.0
12	24,178	117.8	26.1	91.7	2.1
**Post-campaign week**					
13	17,327	0.0	0.0	0.0	0.0
14	19,374	0.0	0.0	0.0	0.0
15	14,614	0.0	0.0	0.0	0.0
16	10,953	0.0	0.0	0.0	0.0

Television GRPs were positively associated with calls to 1–800-QUIT-NOW (Table 1). An increase in 100 GRPs per week for 1–800-QUIT-NOW ads in a given market was associated with an increase of 89 calls per week in a typical area code (β = 89.3, *P* < .001) while the same increase in GRPs for ads tagged with Smokefree.gov was associated with an increase of 29 calls per week (β = 29.4, *P* < .001). The GRP coefficient for ads tagged with 1–800-QUIT-NOW was significantly greater than the GRP coefficient for ads tagged with Smokefree.gov (*P* < .001). A 10% increase in GRPs for ads tagged with 1–800-QUIT-NOW and Smokefree.gov would yield a 2.2% (elasticity = 0.219) and 2.0% (elasticity = 0.196) increase in calls, respectively. The association between radio GRPs and calls to 1–800-QUIT-NOW was smaller but significant (β = 17.4, *P* = .02). We found no association between Hispanic television GRPs and call volume (Table 1).

**Table 1:** Regression Model Results^a^ Showing Relationship Between Weekly Area Code-Level 1–800-QUIT-NOW Call Volume and Weekly Market-Level *Tips* Campaign GRPs, February 4–July 8, 2012

Independent Variable	β Coefficient (*P* Value^b^) [95% CI]	Elasticity^c^	Mean Market-Level Weekly GRP
1-800-QUIT-NOW Television GRP (in 100s)	89.3 (<.001) [72.9 to 105.8]	0.219	33.8
Smokefree.gov television GRP (in 100s)	29.4 (<.001) [23.0 to 35.7]	0.196	87.0
Hispanic television GRP (in 100s)	3.15 (.66) [−10.8 to 17.1]	0.004	15.8
Radio GRP (in 100s)	17.4 (.02) [2.83 to 32.0]	0.004	5.5

Abbreviations: CI, confidence interval; GRP, gross rating point.

a Adjusted *R* ^2^ = 0.74.

b Calculated from standard ordinary least squares regression *t* tests for each regression coefficient.

c Elasticity is the percentage change in calls for a given percentage change in GRPs. Models control for weekly time trend, area code population size, percentage of media market population that is African American, percentage of media market population that is Hispanic, percentage of media market population that has bachelor’s degree or higher degree, media market median income, media market smoking prevalence, state fixed effects, and GRPs for *Tips* ads aired through separate state media buys.

Post-estimation predictions showed that 190,799 (95% confidence interval [CI], 181,084–200,514) calls to 1–800-QUIT-NOW would have been placed in the absence of the *Tips* campaign (ie, if all campaign GRPs equaled zero) during the same 12-week period (Table 2). Based on the 95% CI of this estimate, the difference between this prediction and the actual 363,656 calls placed is 172,857 (95% CI, 163,142–182,572) calls, the number of campaign-attributable calls. We further estimated there would have been 313,522 (95% CI: 301,959–325,085) campaign-attributable calls if all television GRPs had been for ads tagged with 1–800-QUIT-NOW.

**Table 2:** Predicted Calls^a^ Attributable to the *Tips* Campaign for Actual Campaign and Counterfactual Scenario Assuming All Ads Are Tagged With 1–800-QUIT-NOW, February 4–July 8, 2012

Outcome	Prediction Scenario (95% CI)	
	Observed Campaign	Predicted Calls Assuming All Ads Tagged With 1–800-QUIT-NOW
Total calls during *Tips* campaign	363,656	504,321 (492,758–515,884)
Predicted calls in absence of campaign (all GRPs = 0)	190,799 (181,084–200,514)	190,799 (181,084–200,514)
Predicted calls attributable to *Tips* campaign	172,857 (163,142–182,572)	313,522 (301,959–325,085)

Abbreviations: CI, confidence interval; GRP, gross rating point.

a Call prediction values are limited to the 12-week time frame of the *Tips* campaign, March 19–June 10, 2012.

## Discussion

This is the first study to calculate the magnitude of the dose–response of nationwide quitline calls to unit increases in weekly GRPs for national *Tips* campaign ads with quitline and website taglines. As expected, the dose–response effect of ads tagged with 1–800-QUIT-NOW was greater (approximately 89 calls per week per 100 weekly GRPs) than the effect of ads tagged with Smokefree.gov (approximately 29 calls per week). We estimate that the campaign was responsible for more than 170,000 additional calls to 1–800-QUIT-NOW between March 19 and June 10, 2012. These findings provide a national perspective to the evidence base for media campaign effects on quitline call volume in the United States, which, to date, is primarily based on state-specific campaigns (6,8,15).

Our findings have several practical implications for the use of ad taglines. We estimated that the *Tips* campaign would have generated approximately 140,000 additional calls had all ads been tagged with 1–800-QUIT-NOW. This result provides a direct estimate of the potential additional quitline capacity needed at the national level should future campaigns of similar scale use the 1–800-QUIT-NOW tagline exclusively. Future campaigns aimed at expanding quitline use should also examine the costs and required changes in services capacity at the national level to meet additional demand generated by exclusive quitline ad tagging.

We found that although the 1–800-QUIT-NOW tagline had the strongest effect on call volume, ads tagged with Smokefree.gov were also associated with an increase in call volume. Ads tagged with Smokefree.gov may have increased smokers’ interest in quitting and triggered their recall of the quitline from other *Tips* ads tagged with 1–800-QUIT-NOW. Alternatively, some smokers may have gotten the 1–800-QUIT-NOW number directly from the Smokefree.gov website. This suggests that call volume can be promoted indirectly by tagging ads with other information resources. Additionally, perhaps quitline call volume can be managed through ad-tagging strategies without compromising overall ad exposure through reduced ad GRP purchases.

Radio advertising GRPs were associated with smaller increases in calls compared with television advertising GRPs. The smaller magnitude of the radio ads’ effect is probably due to the fact that radio was a smaller component of the *Tips* campaign; only 18 local markets had radio ads. Although radio had smaller effects, radio ads cost less than television ads. Our findings are consistent with at least one other study that demonstrates that radio advertising expenditures are associated with increased calls to quitlines and that the effect of radio advertising is smaller than that of television (14).

This study reinforces evidence that media campaigns have relatively immediate effects on information-seeking behavior, such as calling a help line (14,15,25,26). Trends in calls to 1–800-QUIT-NOW demonstrate rapid increases in calls after the launch of the *Tips* campaign and similarly rapid decreases after the campaign’s conclusion. This high sensitivity to media stimuli suggests that sustained exposure to antismoking messages is required to maintain consistent, increased use of quitlines by smokers. Maintaining broad national exposure to hard-hitting tobacco education campaigns is noted in the recent Surgeon General’s report (27) as an effective evidence-based approach for encouraging smokers to quit. However, maintaining a national ad campaign at a high level over time is challenging given the substantial resources needed. 

Our study has several limitations. First, our data represent total call volume and do not discern call repetition, duration, quality, or other aspects of callers’ interactions with quitline operators and counselors. However, the North American Quitline Consortium endorses total call volume as a measure of promotional reach (5). Second, although we identified the per-GRP dose–response effect of the *Tips* campaign on call volume, we did not attempt to predict the exact levels of GRPs at which call volume is maximized. Further research is needed to better inform recommendations on the size of a media campaign for purposes of promoting quitline calls. Third, our data were limited to call volume to 1–800-QUIT-NOW and did not include call volume for other state quitlines directly promoted in those states. Hence, our findings on the *Tips* campaign effects of calls to 1–800-QUIT-NOW in these states would be lower than the actual effects if *Tips* motivated smokers who knew the alternate numbers to call those numbers instead. Future research should focus on these limitations to better understand the impact of the *Tips* campaign on how callers use quitlines in states that have their own quitline numbers.

Although the *Tips* campaign had a significant impact on nationwide quitline use in 2012, its implementation was brief, spanning only 3 months because of limited resources. CDC’s *Best Practices for Comprehensive Tobacco Control Programs* (1) recommends that tobacco prevention media campaigns run for at least 3 to 6 months to achieve awareness of the issue, 6 to 12 months to have an impact on attitudes, and 12 to 18 months to influence behaviors (1). Although our findings suggest that a longer duration of the *Tips* campaign could have resulted in higher call totals, we could only speculate on the magnitude of such increases because of the lack of data beyond the 12-week campaign. Higher doses or longer durations of campaigns could result in diminishing returns (28–30) and in attenuation of the linear relationship we found. Since the initial 2012 *Tips* campaign aired, longer-duration *Tips* campaigns with new ads were implemented in 2013, 2014, and 2015. Data from these campaigns will allow further evaluation of higher doses and longer durations.

Previous evidence (21) shows that the *Tips* campaign had a substantial impact on nationwide quit attempts by smokers, the primary campaign outcome. However, given overall low levels of quitline use by smokers, most of these quit attempts probably occurred without quitline assistance (5). When used, quitlines increase the likelihood of successfully quitting (2,31,32). Hence, sustained implementation of a national campaign such as *Tips* could translate into significant increases in quitline-assisted cessation and increased quit attempts.
